# Supplementary material for: Estimating total human deaths from rabies: A statistical framework leveraging reports of rabies transmission from deceased organ and tissue donors
Source: PLoS Negl Trop Dis. 2026 Sep 21;20(9):e0014731. doi: 10.1371/journal.pntd.0014731 (PMC13592719; doi:10.1371/journal.pntd.0014731)
Supplement: Supplementary file 6 [file pntd.0014731.s005.zip › Result_B/Ukraine/Figure_S3_Ukraine.pdf]

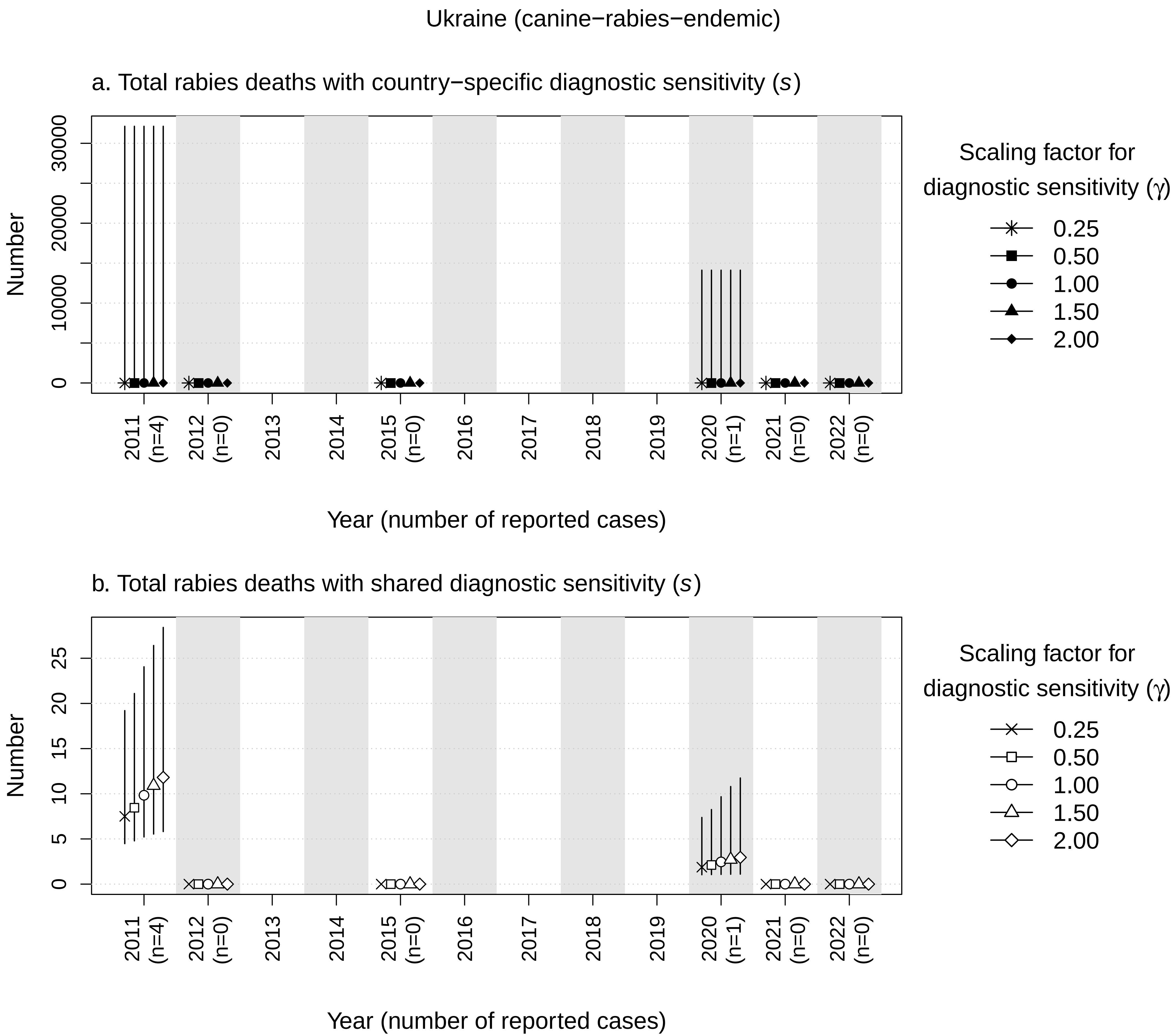

Ukraine (canine−rabies−endemic)
a. Total rabies deaths with country−specific diagnostic sensitivity (s)
Number
0
10000
20000
30000
2011 (n=4)
2012 (n=0)
2013
2014
2015 (n=0)
2016
2017
2018
2019
2020 (n=1)
2021 (n=0)
2022 (n=0)
Year (number of reported cases)
Scaling factor for diagnostic sensitivity (γ)
0.25
0.50
1.00
1.50
2.00
b. Total rabies deaths with shared diagnostic sensitivity (s)
Number
0
5
10
15
20
25
2011 (n=4)
2012 (n=0)
2013
2014
2015 (n=0)
2016
2017
2018
2019
2020 (n=1)
2021 (n=0)
2022 (n=0)
Year (number of reported cases)
Scaling factor for diagnostic sensitivity (γ)
0.25
0.50
1.00
1.50
2.00
